# NF-κB1, c-Rel, and ELK1 inhibit miR-134 expression leading to TAB1 upregulation in paclitaxel-resistant human ovarian cancer

**DOI:** 10.18632/oncotarget.15267

**Published:** 2017-02-11

**Authors:** Ting Shuang, Min Wang, Yingying Zhou, Cong Shi, Dandan Wang

**Affiliations:** ^1^ Department of Obstetrics and Gynecology, Shengjing Hospital of China Medical University, Shenyang 110004, China; ^2^ Department of Obstetrics and Gynecology, Xijing Hospital, The Fourth Military Medical University, Xi’an 710033, China

**Keywords:** miR-134, transcription factor, miRNA gene regulation, ovarian cancer, paclitaxel resistance

## Abstract

The mechanism by which the transcription factors inhibit the miRNA expression in ovarian cancer chemoresistance is unclear. The present study investigated the mechanism underlying the transcriptional repression of miR-134 in chemoresistant serous epithelial ovarian cancer. The results demonstrate that NF-κB1, c-Rel, and ELK1 are involved as transcription factors in repressing miR-134 expression in paclitaxel-resistant ovarian cancer cells. Knockdown of these transcription factors led to increased miR-134 expression, resulting in increased apoptosis and inhibition of proliferation in SKOV3-TR30 cells. NF-κB1, c-Rel, and ELK1 mRNA expression was upregulated in chemoresistant specimens and negatively correlated with miR-134 expression. Kaplan–Meier analysis revealed that high nuclear expressions of NF-κB1, c-Rel, ELK1 were significantly associated with short survival in serous epithelial ovarian cancer patients. Finally, TAB1 was identified as a functional target of miR-134, and the expression of TAB1 was increased by the transcription factors of NF-κB1, c-Rel, and ELK1 via miR-134. Taken together, these results provide an insight into the mechanism of repressed miR-134 expression in chemoresistance of serous epithelial ovarian cancer.

## INTRODUCTION

Ovarian cancer is the most lethal of the gynecological cancers, with approximately 200,000 new cases and more than 100,000 deaths reported every year [1]. Patients routinely undergo a debulking surgery before chemotherapy with Taxol and platinum-based drugs [2, 3]. However, chemoresistance is recurrent, and the 5-year survival rate is approximately 30% [4, 5]. Therefore, the elucidation of the underlying mechanisms in ovarian cancer is an indispensable prerequisite.

MicroRNAs (miRNAs) are small non-coding RNAs (approximately 21–25 nucleotides). In mammalian cells, miRNAs play a significant role in regulating the gene expression at both the transcriptional and post-transcriptional levels [6]. miRNA is also known to cause chemoresistance in ovarian cancer. For example, miR-199b-5p is associated with acquired chemoresistance in ovarian cancer [7] and miR-93 contributes towards cisplatin chemosensitivity in ovarian cancer cells by regulating the PTEN/Akt pathway [8]. Huh et al. [9] found that the dysregulation of miR-106a and miR-591 led to paclitaxel resistance in ovarian cancer while Cittelly et al. [10] discovered that the restoration of miR-200c increased sensitivity to paclitaxel. In a previous study, we showed that decreased miR-134 expression in clinical specimens contributes towards the chemoresistance in serous epithelial ovarian cancer (EOC) patients [11]. Downregulated miR-134 has also been reported in multi-drug-resistant small cell lung cancers and esophageal cancers [12–13].

miRNAs with transcriptions factors (TFs) function in the cancer regulatory networks. For instance, Zhao et al. found that in human gastric cancer, serum response factor expedites metastasis and activates the epithelial-to-mesenchymal transition (EMT) by increased miR-199a-5p expression [14]. Liu et al. demonstrated a Sp1/NF-κB/HDAC/miR-29b regulatory network in myeloid leukemia [15]. Yin et al. showed that hepatocyte nuclear factor-4α regulated the miR-134 expression in the DLK1-DIO3 region leading to the reversal of hepatocellular carcinoma malignancy [16]. However, to our knowledge, the mechanism underlying the transcriptional repression of miR-134 in chemoresistance of ovarian cancer has not yet been reported.

In this study, we identified TFs including NF-κB1, c-Rel, and ELK1 as the factors that might inhibit miR-134 expression and independently render paclitaxel resistance in ovarian cancer cells. We found that NF-κB1, c-Rel, and ELK1 proteins were overexpressed in chemoresistant tissues and were mainly localized in the nucleus. Furthermore, we confirmed the interdependence of mRNA expression of NF-κB1, c-Rel, and ELK1 with miR-134 levels in serous EOC tissues. We also identified TAB1 as a direct target of miR-134 in ovarian cancer cells. Collectively, we report for the first time that the overexpression of NF-κB1, c-Rel, and ELK1, causing the transcriptional repression of miR-134 in ovarian cancer cells, contribute towards their paclitaxel-resistance via miR-134 and that TAB1 might be a direct target of miR-134 in this process. Our study provides a new insight for finding the potential mechanism of ovarian cancer chemoresistance.

## RESULTS

### NF-κB1, c-Rel, and ELK1 repress miR-134 levels in paclitaxel-resistant SKOV3-TR30 ovarian cancer cells

In our previous study, we found that miR-134 expression was decreased in chemoresistant ovarian cancer tissues and paclitaxel-resistant SKOV3-TR30 ovarian cancer cells as compared to that in the chemosensitive tissues and parental SKOV3 cells [11]. In the present study, we aimed to investigate the transcriptional downregulation of miR-134 in ovarian cancer chemoresistance.

**Figure 1:**
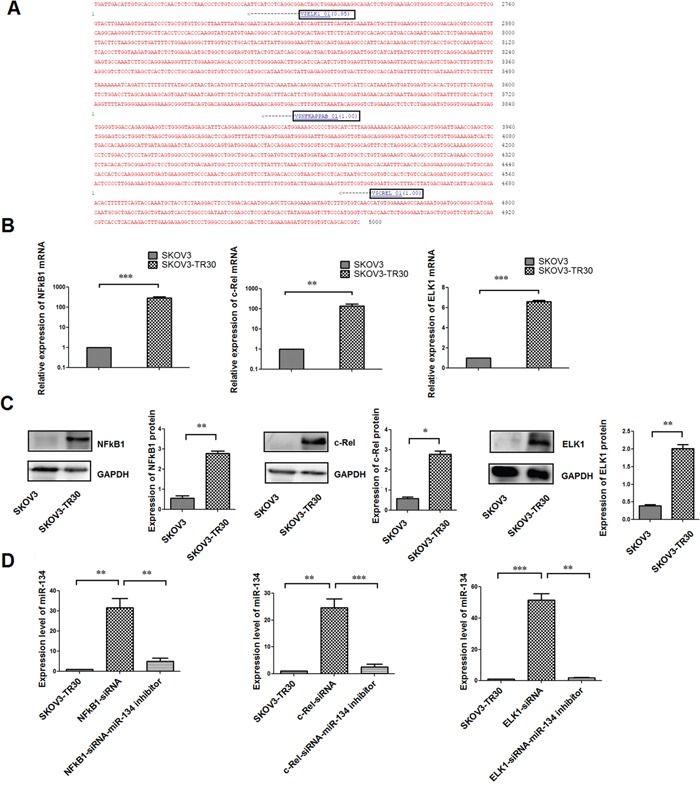
Identification of the functional binding sites in the upstream sequence of pre-miR-134 **A**. The representation of the pre-miR-134 upstream region and the predicted transcription factors are displayed. The region harbors binding sites for various transcription factors and the most likely are shown in black boxes as predicted by TRANSFAC. **B**. Expression of mRNA of the predicted transcription factors NF-κB1, c-Rel and ELK1 in SKOV3 and SKOV3-TR30 cells. **C**. Western blot analysis of the expression of NF-κB1, c-Rel, ELK1 proteins in SKOV3 and SKOV3-TR30 cells. Bar graphs indicate the ratio of the mean densitometry value of the expression of proteins in each group. **D**. The expression of miR-134 decreased significantly after transfection with siRNAs specific for NF-κB1, c-Rel and ELK1. While when cells were co- transfected with siRNA of the TFs NF-κB1, c-Rel and ELK1 along with miR-134 inhibitor, the expression of miR-134 decreased significantly.MiR-134 was normalized against U6. Data represent the mean ± SE of three independent experiments. Data represent the mean ± SE of three independent experiments(*P< 0. 05, **P < 0.01 ***P < 0.001).

In order to identify the potential TFs that might regulate miR-134 expression, we used TRANSFAC® database, the bioinformatics tool [17, 18] to identify and analyze the putative miR-134 promoter region for TF consensus binding sites. Among these predicted TFs, NF-κB1, c-Rel, and ELK1could exert repressive activities of miRNAs as demonstrated in previous studies [19–20]. Moreover, the role of the NF-κB family in the propagation of ovarian cancer cell line has been investigated [21] in addition to the expression of some NF-κB subunits in ovarian cancer tissues [22–24]. Studies involving ELK1 in cervical, endometrial, and liver carcinomas have also been reported in the past decade [25–27]. Therefore, we assessed the involvement of the above three TFs in mediating the repression of miR-134 in ovarian cancer chemoresistance. (Table 1, Figure 1A). Next, we tested the expression of NF-κB1, c-Rel, and ELK1 at both mRNA and protein level in paclitaxel-resistant SKOV3-TR30 cells and the parental SKOV3 cells. The result revealed an overexpression of these TFs at both mRNA (Figure 1B) and protein (Figure 1C) level in SKOV3-TR30 cells as compared to that in the SKOV3 cells.

**Table 1:** Predicted transcription factors and the binding sites in the sequence upstream of pre-miR-134

position (strand)	cored-scored	matrix-score	sequence (always the (+)-strand is shown)	factor name
2816 (-)	1	0.955	agggaCATCCagtttt	ELK1
3900 (-)	1	1	ggaaaGCCCC	NF-κB1
4769 (-)	1	0.997	GGAAAagcca	REL

We also investigated the relevance of these TFs in repressing miR-134 expression by using RNAi technology for the knockdown of NF-κB1, c-Rel, and ELK1 expression respectively. The successful transfection of the NF-κB1, c-Rel, and ELK1 siRNAs was confirmed by qRT-PCR and Western blot analyses (Supplementary Figure 1). Based on the results, we selected siRNA-NF-κB1-2, siRNA-c-Rel-1, and siRNA-ELK1-2 in the subsequent assays. These siRNAs were individually transfected into SKOV3-TR30 cells, and miR-134 expression examined. The transfection of the siRNAs for each of the three TFs resulted in a significant upregulation of miR-134 expression (P = 0.002, P = 0.007 and P < 0.0001, respectively) compared with the levels in the untransfected cells. Conversely, when the cells were co-transfected with siRNA of the TFs NF-κB1, c-Rel, and ELK1 along with the miR-134 inhibitor, the expression of miR-134 decreased significantly (P = 0.005, P < 0.0001, and P = 0.003, respectively) (Figure 1D).

### NF-κB1, c-Rel, and ELK1 transcriptionally repress miR-134 expression by directly targeting the putative miR-134 promoter region in paclitaxel-resistant ovarian cancer cells

**Figure 2:**
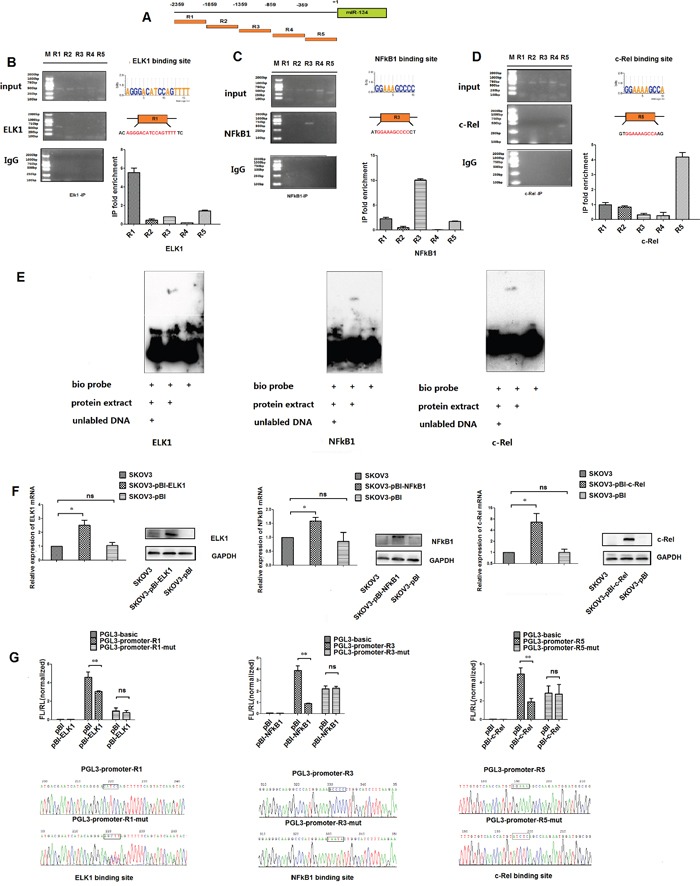
NF-κB1, c-Rel and ELK1 bind directly to the response elements in the putative promoter of miR-134 in ovarian cancer cells **A**. Five genomic regions (R1–R5) spanning a 2.4 kb sequence upstream of the pre-miR-134. **B-D**. Binding of NF-κB1, c-Rel and ELK1 to the miR-134 promoter region was validated in SKOV3-TR30 cells by ChIP. Non-immune IgG and input DNA served as negative and positive controls, respectively. The enrichment of the binding of NF-κB1, c-Rel and ELK1 with the R3, R5 and R1 regions was quantified from the corresponding ChIP with qPCR. **E**. EMSA was performed with nuclear extracts from SKOV3-TR30 cells incubated with 5′-biotin-labeled oligonucleotide sequences containing the binding sites for the NF-κB1, c-Rel and ELK1 transcription factors. Unlabeled competitor sequence was also included to indicate the specificity of the protein-DNA complexes. **F**. pBI- NF-κB1, pBI- c-Rel and pBI- ELK1 overexpression plasmids were transfected into SKOV3 cells; the transfection efficiency was validated by qRT-PCR and Western blot analyses. **G**. At 48 h after transfection, luciferase activity was determined and then normalized to *Renilla* values. **H**. Sequencing was used to identify that the plasmids including pGL3-promoter-R1 (containing the ELK1 binding site) and its mutant pGL3-promoter-R1-mut, pGL3-promoter-R3 (containing the NF-κB1 binding site) and its mutant pGL3-promoter-R3-mut as well as pGL3-promoter-R5 (containing the c-Rel binding site) and its mutant pGL3-promoter-R5-mut were successfully constructed for luciferase reporter assay. Data represent the mean ± SE of three independent experiments(*P< 0. 05, **P < 0.01).

To investigate the mechanism through which NF-κB1, c-Rel, and ELK1 repress the transcription of miR-134, we first conducted chromatin immunoprecipitation (ChIP) with an anti- NF-κB1, anti- Rel, and anti-ELK1 antibody, respectively, to analyzed the specific physical interaction of the TFs with the predicted regions identified. (Figure 2A). Among these regions, the R1, R3, and R5 regions contain the binding sites for ELK1, NF-κB1, and c-Rel, respectively (Figure 2B–2D). A clear band amplified from the ChIP product, immunoprecipitated with specific antibodies, using primers for each region, confirmed that NF-κB1 binds to the R3 region, c-Rel binds to the R5 region, and ELK1 binds to the R1 region. These interactions were confirmed in the SKOV3-TR30 cells by qPCR (Figure 2B–2D). Furthermore, the physical interactions between NF-κB1, c-Rel, and ELK1 and the binding sites within the specific regions were analyzed by electromobility gel shift assay (EMSA). Nuclear proteins extracted from SKOV3-TR30 cells were incubated with biotin-labeled probes (unlabeled probes were used in the competition group). The biotin-labeled probes for NF-κB1, c-Rel, or ELK1 binding sites were able to form complexes with the respective proteins in the nuclear extract. Upon addition of excessive unlabeled oligonucleotide, we observed an abolition of the shifted complex since the excess unlabeled probe is able to compete for the binding sites for complex formation (Figure 2E). Taken together, the results of both ChIP and EMSA analyses in SKOV3-TR30 cells confirmed the physical binding of NF-κB1, c-Rel, and ELK1 to the putative miR-134 promoter. Then, we determined the transcriptional modulating activities of TFs interacting with the corresponding binding sites. In order to achieve this, we cloned the fragment containing these binding sites of ELK1, c-Rel, and NF-κB1, the regions of R1, R3, and R5 fragments upstream of a minimal promoter in a pGL3-promoter luciferase reporter construct to generate pGL3-promoter-R1 (containing the ELK1 binding site), pGL3-promoter-R3 (containing the NF-κB1 binding site), and pGL3-promoter-R5 (containing the c-Rel binding site), respectively. The corresponding mutant binding sites in the pGL3-promoter luciferase reporter construct named them as pGL3-promoter-R1-mut, pGL3-promoter-R3-mut, and pGL3-promoter-R5-mut (Figure 2H) were also generated. We also constructed NF-κB1, c-Rel, and ELK1 overexpression plasmids to investigate the functional regulation of these TFs on the activity of the luciferase reporters. The gene and protein expression levels of NF-κB1, c-Rel, and ELK1 were noticeably increased after the transfection of the overexpression plasmids (Figure 2F). Following co-transfection of SKOV3 cells with pGL3-promoter-R1 with the ELK1 overexpression plasmid pBI-ELK1, the reporter activity decreased significantly as compared to pGL3-promoter-R1 co-transfected with the empty plasmid pBI while in the mutant group, the luciferase activity was similar. Also, the co-transfection of NF-κB1 with pGL3-promoter-R3 and that of c-Rel with pGL3-promoter-R5 resulted in a significant reduction in the luciferase activity as compared to those co-transfected with the empty plasmid pBI, whereas in the mutant group, the luciferase activity was similar (Figure 2G). The luciferase reporter assays in SKOV3 cells showed that the interaction of ELK1, c-Rel, and NF-κB1, with predicted binding sites, could inhibit the activity of miRNA-134 predicted promoter. Taken together, we conclude that NF-κB1, c-Rel, and ELK1 could contribute to the transcriptional inhibition of miR-134 expression.

### NF-κB1, c-Rel, and ELK1 enhances paclitaxel sensitivity and induces apoptosis of SKOV3-TR30 cells by downregulating miR-134

In our previous study, we found that decreased miR-134 expression contributes to paclitaxel-resistance in SKOV3-TR30 ovarian cancer cells. Therefore, we hypothesized that NF-κB1, c-Rel, and ELK1 enhanced the paclitaxel sensitivity of SKOV3-TR30 cells through the repression of miR-134.

**Figure 3:**
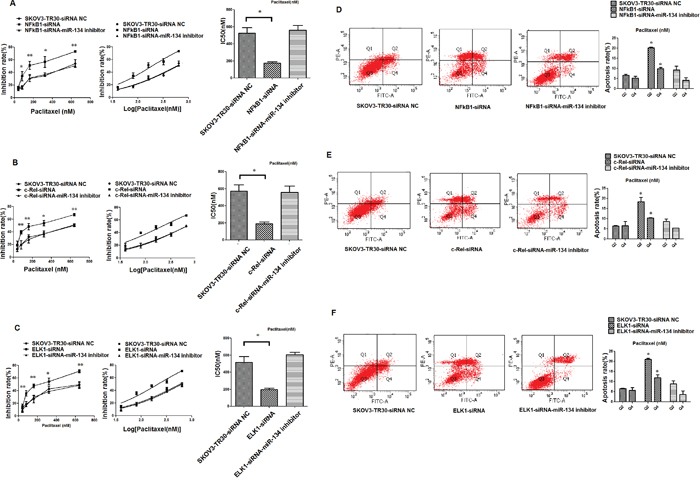
Knockdown of NF-κB1, c-Rel and ELK1 expression stimulates paclitaxel-sensitivity and promotes apoptosis in SKOV3-TR30 cells in the context of miR-134 **A–C**. Cytotoxicity induced by paclitaxel in SKOV3-TR30 cells transfected with siRNA-NF-κB1, siRNA-c-Rel and siRNA ELK1, SKOV3-TR30 cells transfected with the siRNA of TFs NF-κB1, c-Rel and ELK1 along with miR-134 inhibitor (siRNA NC as control group). The cells were treated with paclitaxel at the indicated concentrations for 36 h. Percent survival was determined using the CCK-8 assay. Dose–response curves and IC50 values were generated using GraphPad Prism 5.0. Data represent the mean IC_50_ ± SE of each group, n = 3; *P < 0.05, ** *P* < 0.01. **D–F**. Annexin V/PI apoptosis assay of cells transfected with NF-κB1, c-Rel ELK1-siRNA, cells transfected with the siRNA of TFs NF-κB1, c-Rel and ELK1 along with miR-134 inhibitor and control-siRNA prior to treatment with paclitaxel for 24 h. The relative percentages of live (lower-left quadrant), early apoptotic (lower-right quadrant), late apoptotic (upper-right quadrant) and necrotic (upper-left quadrant) cells are shown. The ratio of apoptosis among different experimental groups was shown in the bar-chart, data represent the mean ± SE of three independent experiments (*P< 0. 05).

To investigate the effect of NF-κB1, c-Rel, and ELK1 on ovarian cancer paclitaxel resistance, we transfected siRNA-NF-κB1-2, siRNA-c-Rel-1, and siRNA-ELK1-2 (siRNA-NC) independently into SKOV3-TR30 cell. After 36 h, the transfected cells were treated with increasing concentrations of paclitaxel (range, 40–640 nM) and the paclitaxel IC50 values were determined by CCK-8 cell survival assays. Subsequent to the treatment with paclitaxel, the IC50 value of SKOV3-TR30-siRNA-NF-κB1-2 cells was significantly higher as compared to that of SKOV3-TR30-siRNA NC cells (525.55 ± 65.65 nM vs.175.5 ± 15.9 nM, P = 0.035) (Figure 3A). The IC50 value of SKOV3-TR30-siRNA-c-Rel-1 cells was also significantly higher than that of SKOV3-TR30-siRNA NC cells (571.8 ± 73.85 nM vs. 190.3 ± 20.3nM (P = 0.038) (Figure 3B). A similar result was observed in the case of the IC50 value of SKOV3-TR30-siRNA-c-ELK1-2 cells when compared with the SKOV3-TR30-siRNA NC cells (516.5 ± 67.9 nM vs. 196.6 ± 17.1 nM, P = 0.045) (Figure 3C). CCK-8 assays showed that the knockdown of NF-κB1, c-Rel, and ELK1, each significantly increased the sensitivity of SKOV3-TR30 cells to paclitaxel, indicating that these TFs could act as vital factors in paclitaxel sensitivity in SKOV3-TR30 cells.

Consequently, we employed a ‘rescue’ experiment by transfecting siRNA- NF-κB1-2 along with miR-134 inhibitor in SKOV3- TR30 cells (transfected with only siRNA-NF-κB1-2 as control), siRNA-c-Rel-1 along with miR-134 inhibitor in SKOV3- TR30 cells (transfected with only siRNA-c-Rel-1 as control), and siRNA- ELK1-2 and miR-134 inhibitor in SKOV3- TR30 cells (transfected with only siRNA- ELK1-2 as control). CCK-8 assay on these transfected cells revealed that the IC50 value was significantly increased in SKOV3-TR30 transfected with siRNA against the TFs (NF-κB1, c-Rel, and ELK1) in the presence of miR-134 inhibitor as compared to the control group. (Figure 3A–3C).

Since anti-apoptosis is known as one of the reasons for the development of paclitaxel resistance, we evaluated the influence of these TFs on apoptosis of SKOV3-TR30 cells by siRNA-mediated knockdown of the TFs (siRNA-NC was used as the control) and analyzed using the Annexin V-FITC/PI apoptosis assay. After transfection, the cells were exposed to paclitaxel for 24 h. SKOV3-TR30 cells transfected with siRNA- NF-κB1-2, siRNA-c-Rel-1, or siRNA-ELK1-2 showed an elevated rate of apoptosis than the siRNA NC-transfected cells. The cells transfected with siRNA of siRNA-NF-κB1-2, siRNA-c-Rel-1, and siRNA-ELK1-2 along with miR-134 inhibitor displayed a decreased rate of apoptosis than the cells transfected with the only siRNA of TFs (Figure 3D-3F). These results indicated that NF-κB1, c-Rel, and ELK1 suppress the apoptosis and ovarian cancer cells’ sensitivity to paclitaxel via miR-134.

### NF-κB1, c-Rel, and ELK1 overexpression negatively correlates with miR-134 decreased expression in serous EOC specimens and are strong risk factors associated with chemoresistance and the prognosis of serous EOC

**Figure 4:**
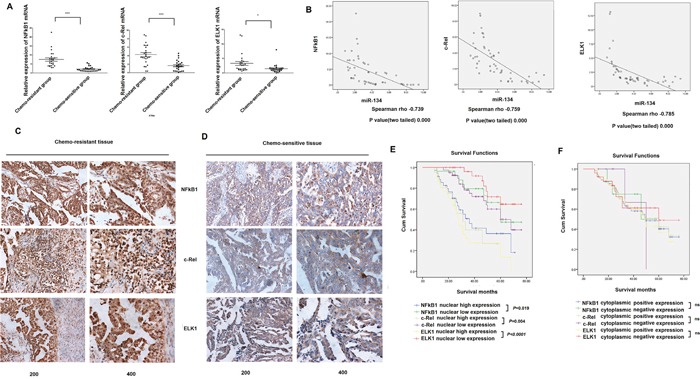
Correlations between miR-134 levels and expression of NF-κB1, c-Rel and ELK1 in serous EOC specimens **A**. NF-κB1, c-Rel and ELK1 mRNA expression was analyzed by qRT-PCR in tissue from 24 cases of chemosensitive serous EOC and from 24 cases of chemoresistant serous EOC. NF-κB1 mRNA expression was significantly upregulated in chemoresistant serous EOC tissues, while c-Rel mRNA and ELK1 mRNA were overexpressed in chemoresistant tissues compared with that in chemosensitive tissues. **B**. Spearman correlation coefficient analyses of the correlations between miR-134 and expression of NF-κB1, c-Rel and ELK1 were performed for data obtained from qRT-PCR results and the correlation coefficient ‘r’ was calculated. **C-D**. Immunohistochemical staining of NF-κB1, c-Rel and ELK1 in serous epithelial ovarian cancer tissues. C, Expression of NF-κB1, c-Rel and ELK1 in chemoresistant tissue. D, Expression of NF-κB1, c-Rel and ELK1 in chemosensitive tissue. **E**. Patients with high nucleus NF-κB1, c-Rel and ELK1 expression showed significantly shorter overall survival than those with low nucleus expression. **F**. The positive cytoplasmic expression of NF-κB1, c-Rel and ELK1 showed no effect on survival in serous EOC patients.

The expression of NF-κB1, c-Rel, and ELK1 in tissue samples obtained from serous EOC patients (information about clinical specimens is shown in Supplementary Table 1) was analyzed by qRT-PCR following extraction of RNA. NF-κB1, c-Rel, and ELK1 mRNA was significantly overexpressed in chemoresistant tissues compared with the chemosensitive tissues (7.497 ± 0.9112 vs. 2.175 ± 0.276, P < 0.0001; 4.258 ± 0.480 vs. 1.655 ± 0.251, P < 0.0001; and 3.319 ± 0.682 vs. 1.492 ± 0.316, P = 0.0056, respectively) (Figure 4A). Our previous study showed that miR-134 levels were decreased in serous EOC tissues and that decreased miR-134 expression correlated with serous EOC chemoresistance [11]. Therefore, we evaluated the correlations of NF-κB1, c-Rel, and ELK1 with miR-134 expression in clinical tissue samples obtained from serous EOC patients (the miR-134 expression in serous EOC specimens was tested in our previous study). Spearman's correlation analysis revealed significantly negative correlations between miR-134 and NF-κB1 (r = -0.739, P < 0.0001), c-Rel (r = -0.759, P < 0.0001), and ELK1 (r = -0.785, P < 0.0001) (Figure 4B). These data indicated a statistically significant dependence of miR-134 expression on the TFs, NF-κB1, c-Rel, and ELK1.

Furthermore, immunohistochemistry (IHC) analysis showed that NF-κB1, c-Rel, and ELK1 were overexpressed in chemoresistant EOC tissues compared with the chemosensitive tissues. Moreover, the staining was predominantly observed in the nucleus in the chemoresistant tissues (Figure 4C–4D). Correlation analysis revealed that the nuclear expressions of NF-κB1, c-Rel, and ELK1 were significantly associated with chemotherapy resistance in serous EOC (Table 2). Kaplan–Meier analysis further demonstrated that high nuclear expressions of NF-κB1, c-Rel, and ELK1 were significantly associated with a shorter survival rate in serous EOC patients (Figure 4E), whereas the cytoplasmic expression of the TFs did not interfere with the survival of serous EOC patients (Figure 4F). Furthermore, multivariate Cox regression analysis indicated that high nuclear expressions of ELK1 and c-Rel as well as the FIGO stage served as independent prognostic factors for poor survival in serous EOC patients (Table 3).

**Table 2:** Correlation of NF-κB1, c-Rel and ELK1 expression with patients’ clinicopathological variables in tissue of EOC

Clinical pathologic factors		NFkB1 nuclear expression		c-Rel nuclear expression			ELK1 nuclear expression			
	total	Low(-/+)	High(++/+++)	P	Low(-/+)	high (++/+++)	P	low(-/+)	high (++/+++)	P
	n	case	case		case	case		case	case	
	63									
**Differentiation**				0.004			0.932			0.839
High	5	3	2		3	2		3	2	
Moderate	36	9	27		23	13		17	19	
Poorly	22	15	7		13	9		10	12	
**FIGO stage**				0.623			0.038			0.001
I-II	14	6	8		12	2		12	2	
III-IV	49	21	28		27	22		18	31	
**lymph node metastasis**				0.277			0.035			0.036
Detected	16	5	11		6	10		4	12	
Not detected	47	22	25		33	14		26	21	
**Chemo-sensitivity**				<0.0001			<0.0001			<0.0001
chemo-sensitive	35	22	13		29	6		29	6	
chemo-resistant	28	5	23		10	18		2	26	

**Table 3:** Multivariate analysis of prognosis in serous EOC patients

Type	B	SE	Sig.	Exp(B)	95.0% CI	
**NFkB1**	-0.308	0.536	0.565	0.735	0.257	2.099
**c-Rel**	-1.092	0.505	0.031	0.336	0.125	0.903
**ELK1**	-1.946	0.55	0.0001	0.143	0.049	0.42
**Differentiation**	-0.431	0.505	0.393	0.65	0.242	1.748
**FIGO stage**	1.168	0.413	0.005	3.215	1.432	7.218
**lymph node metastasis**	0.694	0.537	0.197	2.001	0.698	5.733

### TAB1 is a direct target of miR-134 in ovarian cancer cells that overexpressed in chemoresistant tissues

**Figure 5:**
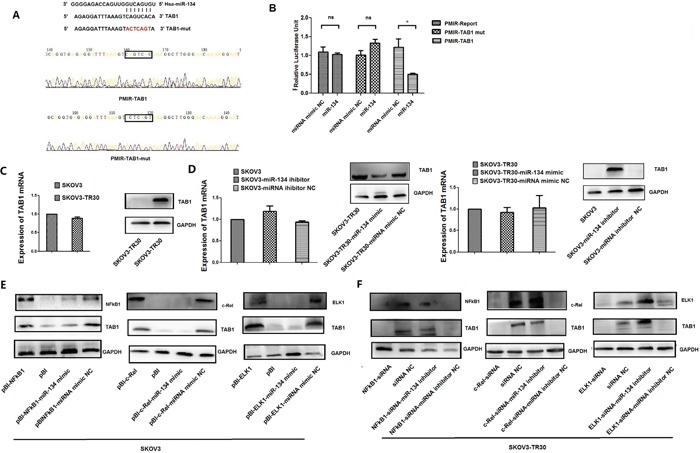
TAB1 is a direct target of miR-134 in ovarian cancer cells **A**. The putative TAB1 binding site was identified by target gene prediction software (TargetScan, miRDB, and miRanda databases). The successful construction of the PMIR-TAB1 and PMIR-TAB1-mut plasmids for luciferase reporter assays was confirmed by sequencing. **B**. Luciferase reporter assays were performed to confirm the direct interaction of miR-134 with the TAB1 binding site sequences. SKOV3-TR30 cells were co-transfected with miR-134 mimic or miRNA mimic NC and the luciferase reporter constructs (PMIR- TAB1), the mutant (PMIR- TAB1-mut), or PMIR-Report. The assays were performed in triplicate (*P < 0.05, “ns” no significant difference). **C-D**. MiR-134 regulation of TAB1 expression at the post-transcriptional level. TAB1 mRNA expression in SKOV3 and SKOV3-TR30 cells was analyzed by real-time PCR. GAPDH was used as an internal control. TAB1 protein levels were analyzed by Western blotting. GAPDH was used as an endogenous loading control. **E-F**. NF-κB1, c-Rel, and ELK1 lead to the upregulation of TAB1 via miR-134. Western blot estimated the TAB1 protein levels in SKOV3-TR30 and its parental SKOV3 cells after transfection.

We used computational methods (TargetScan, miRDB, and miRanda databases) to identify the potential targets of miR-134. Among these targets, we focused on *TAB1*, which was predicted as a target gene of miR-134 by all the three databases (the other overlapping target genes of miR-134 are shown in Supplementary Table 3). Importantly, TAB1 binds to and activates TAK1, suppresses pro-apoptotic signaling pathways, and thus, promotes resistance to chemotherapeutic drugs [28–30]. However, whether TAB1 is effective in inducing chemoresistance to therapeutic drugs against ovarian cancer is not yet clearly understood. First, to determine whether TAB1 is a direct target of miR-134, we constructed reporter plasmids using wild-type or mutant TAB1 3′-UTR fragments (Figure 5A). As miR-134 expression was decreased in SKOV3-TR30 cells compared with that in the SKOV3 cells, we transfected SKOV3-TR30 cells with miR-134 mimic and the pMIR-TAB1 reporter. Luciferase reporter assay showed that the co-transfection of pMIR-TAB1 with miR-134 mimic led to a significant decrease in the luciferase activity compared with the co-transfection of pMIR-TAB1 with miRNA mimic NC (0.499 ± 0.033 vs. 1.218 ± 0.221, P = 0.032). Contrastingly, the pMIR-TAB1-mutant reporter and the empty pMIR reporter groups did not show any significant differences in the luciferase activity when co-transfected with the miR-134 mimic and the miRNA mimic NC (Figure 5B). Real-time PCR assays showed that neither the overexpression nor the inhibition of miR-134 altered the TAB1 mRNA levels (Figure 5C-5D). However, Western blot analysis revealed that miR-134 overexpression in SKOV3-TR30 cells significantly suppressed TAB1 protein levels, whereas miR-134 inhibition in SKOV3 cells increased the TAB1 protein expression (Figure 5C-5D). These observations indicated that miR-134 downregulates TAB1 expression at the post-transcriptional level. Intriguingly, by over-expressing the TFs (NF-κB1, c-Rel, and ELK1) in SKOV3 cells and consecutively increasing the miR-134 expression by transfecting miR-134 mimic or downregulating these TFs along with decreasing the expression of miR-134 by transfecting miR-134 inhibitor in SKOV3-TR30 cells, we found that the expression of TAB1 could be up-reuglated by the TFs, NF-κB1, c-Rel, and ELK1 via miR-134 (Figure 5E-5F).

**Figure 6:**
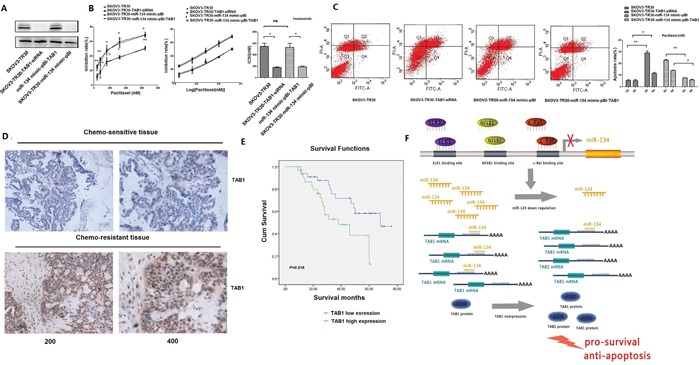
TAB1 contributes to paclitaxel resistance in ovarian cancer cells and is overexpressed in chemoresistant EOC specimens **A**. After transfection, TAB1 protein levels were analyzed by western blotting. **B**. CCK-8 results showed that TAB1 siRNA treatment caused increased cell inhibition rate in SKOV3-TR30 cells at 36 h, and the IC50 value was significantly lower than the control group. The rate of inhibition of proliferation decreased significantly in SKOV3-TR30 cells transfected with miR-134 mimic in combination with pBI-TAB1 compared with that in SKOV3-TR30 cells transfected with miR-134 mimic in combination with pBI empty vector. Data are represented as mean ± SE of three independent experiments (*P < 0.05, **P < 0.01). **C**. Flow cytometric analysis of apoptosis in these groups. Results showed that when TAB1 expression was suppressed, cell apoptosis increased significantly. The rate of apoptosis in cells transfected with miR-134 mimic combined with pBI-TAB1 was decreased compared with that in the cells transfected with miR-134 mimic combined with pBI empty vector. Data are represented as mean ± SE of three independent experiments (*P < 0. 05, **P < 0.01). **D**. IHC staining for detection of TAB1 expression in serous EOC tissues. **E**. Kaplan–Meier analysis indicated that the survival rate was significantly decreased in the TAB1 high-expression group compared with that in the low-expression group (P=0.016). **F**. Model of the NF-κB1, c-Rel, and ELK1 inhibiting the miR-134 expression leading to TAB1 upregulation in ovarian cancer paclitaxel-resistant cells.

Then, we tested whether RNAi-mediated reduction of TAB1 level increase the cell inhibition and promote apoptosis of SKOV3- TR30 cells. The protein levels of TAB1 decreased significantly after SKOV3-TR30 treated with TAB1 siRNA (Figure 6A). To further investigate the effect of TAB1 on cell proliferation and paclitaxel resistance, SKOV3-TR30 cells transiently transfected with siRNA of TAB1 (siRNA-NC) were treated with increasing paclitaxel concentrations, and their inhibition rates and the paclitaxel IC50 values were determined by CCK-8 cell survival assays. The results showed that TAB1 siRNA treatment caused an increased rate of cell inhibition in SKOV3-TR30 cells at 36 h, and the IC50 value was significantly lower than the control group (179.2±10.0 nM vs. 550.0 ± 55.5 nM, P = 0.022) (Figure 6B). The flow cytometry analysis showed that when TAB1 expression was suppressed, cell apoptosis was increased significantly (Figure 6C).

To clarify whether the augmented TAB1 levels might account for the increase in cell survival and decreased apoptosis associated with low miR-134 expression, we performed a “rescue” experiment by co-transfecting SKOV3- TR30 cells with the miR-134 mimic and the TAB1 overexpression plasmid, pBI-TAB1 (transfected with miR-134 mimic combined with pBI as an internal control). The CCK-8 assay showed that the inhibition of proliferation was decreased significantly in SKOV3-TR30 cells transfected with miR-134 mimic coupled with pBI-TAB1 as compared to the inhibition observed in SKOV3-TR30 cells transfected with miR-134 mimic combined with the pBI empty plasmid. The paclitaxel IC50 value was significantly higher than that in the control group (534.5 ± 66.7 vs. 191.0 ± 14.6, P = 0.037) (Figure 6B). Furthermore, cells co-transfected with the miR-134 mimic and the pBI-TAB1 displayed a decreased apoptosis compared with the cells co-transfected with the miR-134 mimic and the pBI empty plasmid (Figure 6C). These results indicate that TAB1 is a direct target of miR-134 in activate cell survival and suppress apoptosis in SKOV3-TR30 cells.

We further investigated the clinicopathological and prognostic significance of TAB1 in serous EOC patients. IHC analysis showed that TAB1 was significantly upregulated in chemoresistant EOC tissues compared with the chemosensitive EOC tissues and was predominantly localized in the cytoplasm (Figure 6D). A correlation analysis revealed that high TAB1 expression was significantly associated with chemoresistance (P = 0.002, Table 4). Kaplan–Meier analysis showed that TAB1 overexpression was associated with a significant decrease in the mortality rate (Figure 6E). Together, these results provide sufficient evidence to conclude that TFs of NF-κB1, c-Rel, and ELK1 transcriptionally repress miR-134 expression, thereby leading to TAB1 upregulation and contributing to ovarian cancer chemoresistance (Figure 6F).

**Table 4:** Correlation of TAB1 expression with patients’ clinicopathological variables in tissue of EOC

Clinical pathologic factors	TAB1 expression			*P*
	All cases	low(-/+)	High(++/+++)	
	n	case	case	
	63	38	25	
**Differentiation**				0.535
High	5	4	1	
Moderate	36	20	16	
Poorly	22	14	8	
**FIGO stage**				0.113
I-II	14	11	3	
III-IV	49	27	22	
**lymph node metastasis**				0.135
Detected	16	7	9	
Not detected	47	32	15	
**Chemosensitivity**				0.002
chemo-sensitive	35	27	8	
chemo-resistant	28	11	17	

## DISCUSSION

In our previous study, we found decreased miR-134 expression in serous EOC tissues contributed towards paclitaxel-resistance in ovarian cancer cells [11]. Recently, the TFs that regulate miRNA transcription have become a focus of intensive research. However, the mechanism underlying the transcriptional repression of miR-134 in ovarian cancer chemoresistance is yet unknown.

In the present study, we used the bioinformatics tool, the TRANSFAC® database [17, 18], to analyze the putative miR-134 promoter region for TF consensus binding sites and identify the binding sites for NF-κB1, c-Rel, and ELK1. We also observed that the NF-κB1, c-Rel, and ELK1 expression was significantly upregulated both at mRNA and protein levels in paclitaxel-resistant SKOV3-TR30 ovarian cancer cells than the parental SKOV3 cell line (Figure 1). Furthermore, siRNA-mediated knockdown of NF-κB1, c-Rel, and ELK1 expression resulted in an upregulated miR-134 expression in SKOV3-TR30 cells. EMSA and ChIP studies confirmed the physical association of NF-κB1, c-Rel, and ELK1 with their respective recognition sites in the putative promoter region of miR-134. Luciferase reporter assays and site-directed mutagenesis studies confirmed that NF-κB1, c-Rel, and ELK1 repress the expression of miR-134 in SKOV3-TR30 cells by inhibiting the transcriptional activities at these sites. In combination, these results demonstrate that NF-κB1, c-Rel, and ELK1 bind to their respective recognition sites in the putative promoter region of miR-134 and repress its expression in paclitaxel-resistant SKOV3-TR30 ovarian cancer cells. Regulation of miRNA genes by direct physical binding of NF-κB p65/RelA has been shown previously for several miRNAs. For instance, p53 and NFκB p65/RelA have been shown to play significant roles in the development of head and neck squamous cell carcinoma by interacting with miR-21 and miR-34a/c [31]. Another study showed that NF-κB p65/RelA directly binds and induces the transcription of miRNAs such as miR-146a, -125b, -9, -155, -21, -221, -222, but suppresses the expression of the miR-199a/214 cluster [32]. Zhang et al. suggested that ELK1 is a transcriptional repressor of miRNA-200b [20]. In the present study, we observed for the first time that NF-κB, c-Rel, and ELK1 bind to their respective recognition sites in the putative promoter region of miR-134 and suppress its expression in paclitaxel-resistant SKOV3-TR30 ovarian cancer cells.

Following siRNA-mediated knockdown of NF-κB1, c-Rel, and ELK1, a significant inhibition of cell proliferation and increased apoptosis was observed. Contrastingly, the cells transfected with siRNA for TFs NF-κB1, c-Rel, and ELK1 along with miR-134 inhibitor displayed significantly decreased cell inhibitory rate and decreased apoptosis (Figure 4D-4F). These results indicated that NF-κB1, c-Rel, and ELK1 exert an impact on apoptosis and sensitivity to paclitaxel in ovarian cancer cells via miR-134. The importance of our findings depends on the pro-survival and anti-apoptotic functions of NFκB1, c-Rel, and ELK1 that are mediated predominantly via decreased miR-134 expression.

High levels of NF-κB1, c-Rel, and ELK1 mRNA were found in clinical specimens of serous EOC chemoresistant tissues, and we identified strong negative correlations between the expression of these TFs at the mRNA level and miR-134 expression in serous EOC tissues. Immunohistochemical studies confirmed that NF-κB1, c-Rel, and ELK1 are over-expressed, mainly in the nucleus, of the chemo-resistant EOC tissues compared with the pattern of expression in the chemosensitive tissues. Cox's regression analysis indicated that FIGO stage and nuclear expression of ELK1 and c-Rel are independent risk factors that correlate strongly with the prognosis of ovarian cancer.

Moreover, to our knowledge, our study provides the first evidence that TAB1 is a direct target of miR-134 in ovarian cancer. Furthermore, our investigations revealed overexpression of TAB1 in chemoresistant EOC specimens and its predominant localization in the cytoplasm. Moreover, the cytoplasmic TAB1 overexpression was shown to be an independent risk factor for chemoresistance in serous EOC. In accordance with our results, Zhu et al. found that TAB1 expression in non-small cell lung carcinoma (NSCLC) tissue is significantly increased and closely associated with patient clinical prognosis [33]. Interestingly, TAB1 as a target of miR-134 involved in the stress response signaling through TGF-B/TAK1/TAB1 and subsequent activation of JNK and p38 kinases might promote the phosphorylation of ELK1 [34]. Furthermore, TAB1 might play a role in NF-κB signaling [35, 36], which also suggests a feedback mechanism regulating the expression of miR-134. Our future studies will persist to unearth the possible feedback mechanism involved in the repression of miR-134 in paclitaxel-resistance of ovarian cancer.

In conclusion, for the first time, we identify the TFs responsible for the decreased expression of miR-134 associated with chemoresistance in ovarian cancer. We also demonstrated that NF-κB1, c-Rel, and ELK1 function by interacting with the binding sites in the putative promoter of miR-134 and repress its expression in SKOV3-TR30 cells. Also, the pro-survival and anti-apoptosis functions of NFκB1, c-Rel, and ELK1 are predominantly mediated by decreased miR-134 expression by these TFs. Furthermore, we found strong negative correlations between the expressions of NF-κB1, c-Rel, ELK1 and miR-134 in clinical specimens of serous EOC tissues. In addition, we clarified that TAB1 is a direct target of miR-134. Thus, our findings elucidate the mechanism by which miR-134 expression is repressed in serous EOC, and provide a new insight into the machinery of chemoresistance in serous EOC.

## MATERIALS AND METHODS

### Tissue samples

The mRNA expression was analyzed in fresh clinical specimens from 48 patients with serous EOC. Tissue samples were obtained during routine surgery at Shengjing Hospital of China Medical University between 2010 and 2012. Any of the patients did not receive radiotherapy or chemotherapy prior to surgery. The patients with progressive disease during primary chemotherapy or those who suffered recurrent disease within 6 months of completing the primary chemotherapy were classified as drug-resistant. The patients who either showed disease recurrence beyond 6 months or no recurrence were classified as drug-sensitive. The present study was approved by the Institutional Review Board of Shengjing Hospital of China Medical University (ethical review approval document number: 2012PS57K).

### Cell culture

The human ovarian carcinoma cell line, SKOV3 and paclitaxel-resistant SKOV3-TR30 cells were provided by the Tumor Cell Bank Research Institute of the Chinese Academy of Medical Sciences, Beijing, China. The paclitaxel-resistant SKOV3-TR30 cells provided by Zhejiang University Affiliated Obstetrics and Gynecology Hospital was derived from SKOV3 cell line by exposing the parental SKOV3 cells to increased concentration of paclitaxel. SKOV3-TR30 cells demonstrated a 27.5-fold greater resistance to paclitaxel than the SKOV3 cells [37]. Both the cell lines were cultured in RPMI 1640 medium (Hyclone, Logan, UT, USA) supplemented with 10% fetal bovine serum (FBS; Gibco Life Technologies, Grand Island, NY, USA) and 1% penicillin/streptomycin (Hyclone). SKOV3-TR30 cells were maintained in the presence of 30 nmol/L of paclitaxel (Sigma-Aldrich, St. Louis, MO, USA). Paclitaxel was withdrawn 1 week before the experiment. All cells were maintained in a humidified atmosphere containing 5% CO_2_ at 37°C. Cells in the logarithmic phase of growth were used in all the experiments.

### Gene overexpression plasmids and luciferase reporter vectors

The genes for the overexpression of NF-κB1, c-Rel, ELK1, and TAB1 were inserted into the pBI plasmid (Clontech). All the restriction enzymes used in this study were fast-digest enzymes. Specifically, the whole CDS region of the *NF-κB1*, *c-Rel*, *ELK1*, and *TAB1* genes was cloned using primers shown in Supplementary Table 2. The PCR products were cleaved with *Mlu* I and *Hind* III, and inserted into the linearized pBI plasmid to obtain the overexpression plasmid constructs, designated as pBI- NF-κB1, pBI- c-Rel, pBI- ELK1, and pBI- TAB1.

We divided the upstream sequence of pre-miR-134 into five regions (R1, R2, R3, R4, and R5). EMSA and ChIP assays were used to confirm that NF-κB1 binds to the R3 region, c-Rel binds to the R5 region and ELK1 binds to the R1 region. Therefore, we cloned the R1, R3, and R5 regions into the pGL3-promoter vector (Promega, Madison, WI, USA) containing the luciferase reporter gene to obtain the pGL3-promoter-R1, pGL3-promoter-R3, and pGL3-promoter-R5 reporter constructs. The R1, R3, and R5 regions of the sequence upstream of pre-miR-134 were generated by PCR using genomic DNA from SKOV3-TR30 cells as a template (primers are listed in the Supplementary Table 2). The PCR product was ethanol precipitated, digested, and then cloned into the *Mlu* I and *Xho* I restriction sites on the pGL3-promoter vector. A site-directed gene mutagenesis kit (ThermoFisher Scientific, Waltham, MA, USA) was then utilized to create the mutant counterparts of the luciferase reporter vectors containing the R1, R3, and R5 regions. The mutant primers for the NF-κB1, c-Rel, and ELK1 binding sites in the R3, R5 and R1 regions, respectively, are also shown in Supplementary Table 2. The mutant plasmids were designated as pGL3-promoter-R1-mut, pGL3-promoter-R3-mut, and pGL3-promoter-R5-mut, respectively.

In order to construct the miRNA 3′-UTR luciferase reporter vectors, the wild-type 3′-UTR of TAB1, containing the putative miR-134 binding sites, was amplified by PCR using the genomic DNA from SKOV3-TR30 cells as a template. The PCR products were cleaved with restriction enzymes, *Spe* I and *Hind* III, prior to insertion into the linearized pMIR reporter vector (Ambion, Carlsbad, CA, USA) to obtain a luciferase reporter construct. The mutant counterparts were constructed using the kit, as described above. All the constructs were verified by sequencing. SKOV3-TR30 cells were utilized in the luciferase activity assays.

### Cell transfection

The pBI- NF-κB1, pBI- c-Rel, and pBI- ELK1 overexpression plasmids were transfected into SKOV3 cells using Lipofectamine 3000 (Invitrogen) according to the manufacturer's instructions for the luciferase reporter assays.

The siRNAs against NF-κB1, c-Rel, ELK1, and TAB1 were designed and synthesized by Sigma (Sigma-Aldrich), followed by transfection into the cells at a final concentration of 50 nM using Lipofectamine 3000 (Invitrogen) according to the manufacturer's protocol (The sequence for each siRNA is shown in Supplementary Table 2).

MiR-134 mimic and miR-134 inhibitor were chemically synthesized by RiboBio (Guangzhou, China). RNA oligonucleotides were transfected into the cells at a final concentration of 100 nM using Lipofectamine 3000 according to the manufacturer's protocol.

### RNA extraction and quantitative real-time polymerase chain reaction (qRT-PCR)

Total RNA was isolated using TRIzol reagent (Invitrogen) according to the manufacturer's instructions. cDNA was synthesized for the detection of NF-κB1, c-Rel, and ELK1 expression using the PrimeScriptVR ^RT^ Reagent Kit (Takara, Dalian, China). A qRT-PCR assay was performed to evaluate the mRNA expression using the SYBR® Select Master Mix (Applied Biosystems Life Technologies, Beijing, China) on the ABI PRISM 7300 Sequence Detection System (Applied Biosystems Life Technologies). GAPDH mRNA was employed as an endogenous control and relative expression levels in each sample were measured by the 2^−ΔΔCT^ method. The primers for the analysis of NF-κB1, c-Rel, ELK1, and TAB1 expression are shown in Supplementary Table 2. For qRT-PCR analysis of miR-134 levels, cDNA was synthesized from 10 ng of total RNA using TaqMan^TM^ miRNA hsa-miR-134 specific primers (Applied Biosystems Life Technologies) and a TaqMan^TM^ microRNA Reverse Transcription kit (Applied Biosystems Life Technologies). All reactions were performed in triplicate.

### ChIP assay

SKOV3-TR30 cells (2 × 10^6^) were plated in 10 cm dishes. After 24 h, the cells were cross-linked and processed according to the Pierce− Agarose ChIP Kit (#26156; Thermo Fisher Scientific). ChIP grade antibodies specific for NF-κB1, c-Rel, and ELK1 (Abcam) were used for immunoprecipitation. Ten percent of chromatin prior to immunoprecipitation was used as input controls, and nonspecific antibody (rabbit anti-IgG) served as the negative control. The extracted DNA was subjected to PCR (30 cycles at 95°C for 45 s, 45°C for 45 s, 72°C for 1 min) with the primers designed to amplify the 5 regions upstream of miR-134 (primers are shown in Supplementary Table 2). The PCR products were separated by agarose gel electrophoresis to visualize the presence of the miR-134 promoter regions. Moreover, the qPCR analysis was used to detect the NF-κB1, c-Rel, and ELK1 binding sites in the region upstream of miR-134. 2 μL ChIP DNA was used as a template and the results were normalized by 1% input for each sample. At least three independent experiments were performed.

### EMSA

EMSA was performed using the LightShift Chemiluminescent EMSA kit (#20148) according to the manufacturer's instructions (Thermo Fisher Scientific). In the present study, the nuclear proteins were extracted from SKOV3-TR30 cells. The nuclear extracts of the SKOV3-TR30 cells were prepared using the NE-PER nuclear and cytoplasmic extraction reagent (Thermo Fisher Scientific). The biotin-labeled oligonucleotides specific for the putative binding sites on NF-κB1, c-Rel, and ELK1 were synthesized (Supplementary Table 2) and annealed. Double-stranded biotin-labeled oligonucleotides were incubated with the nuclear extract for 30 min at room temperature. The subsequent protocol was followed according to the LightShift Chemiluminescent EMSA kit. Unlabeled oligonucleotides were used in the competition experiments. At least three independent experiments were performed.

### Luciferase assays

Cells were transfected with pGL3–R3–promoter and its mutant pGL3–R3–promoter-mut together with pBI- NF-κB1 (pBI), pGL3–R5–promoter and its mutant pGL3–R5–promoter–mut together with pBI-c-Rel (pBI), and pGL3–R1–promoter and its mutant pGL3–R1–promoter–mut along with pBI-ELK1 (pBI) using Lipofectamine 3000. After transfection, the cell lysates were prepared using the Dual-Luciferase® Reporter Assay Kit (Promega) and the luciferase activity was analyzed using the Dual-Luciferase Reporter Assay System (Promega). The transfected cells were analyzed in triplicate for each group.

For 3′UTR reporter assays, cells were plated in a 24-well plate (2×10^5^ cells/well) 24 h before transfection with luciferase reporter constructs, PMIR-TAB1, PMIR-TAB1-mut (400 ng) and miR-134 mimic or miRNA mimic NC (100 nM) using Lipofectamine 3000 according to the manufacturer's instructions. The pRL-TK vector (Promega) was co-transfected as an internal control to optimize the differences in the efficiencies of both transfection and harvesting. After 24 h, the cell lysates were prepared using Dual-Luciferase Reporter Assay kit and luciferase activity was analyzed using the Dual-Luciferase Reporter Assay System. The transfected cells were analyzed in triplicate for each group.

### Western blot analysis

Proteins were harvested using RIPA lysis buffer (Thermo Fisher Scientific), diluted with an SDS-buffer (Life Technologies), and denatured at 95°C for 10 min. The proteins (20 μg) were resolved by SDS-polyacrylamide gel (10%) electrophoresis and transferred to PDVF membranes. The membranes were washed with Tris-buffered saline (TBS, Sigma-Aldrich), blocked with 5% dried non-fat milk (Bio-Rad Laboratories, Hercules, CA, USA) in 1% Tween–TBS. Subsequently, the membranes were incubated overnight at 4°C with rabbit antibody of NF-κB1, c-Rel, ELK1, rabbit anti-TAB1 antibody (Abcam, 1:500), and mouse anti-GAPDH antibody (Abcam, 1:5000), respectively followed by incubation for 1 h at room temperature with a horseradish peroxidase-conjugated detection antibody (1:2000). The immunoreactive bands were visualized with an electrochemiluminescence system and quantified using Chemi-Doc XRS imaging software (Bio-Rad).

### IHC

The paraffin-embedded histological sections from 63 patients with serous EOC, diagnosed at the Shengjing Hospital of China Medical University between 2008 and 2013. Specific antibodies for the detection of NF-κB1, c-Rel, ELK1, and TAB1 (Abcam) were utilized. The results were evaluated by 2 independent observers to control for variability. The expression levels were visualized and classified based on the percentage of positively-stained cells and the staining intensity.

### CCK-8 assay

To evaluate the sensitivity of SKOV3-TR30 cells to paclitaxel after transfection, the cells were seeded in 96-well plates (5 × 10^3^/well) for 24 h before adding paclitaxel (Sigma) at various concentrations. After 36 h incubation, the cell viability was assessed using the CCK-8 assay (Dojindo, Kumamoto, Japan). The absorbance of each well was estimated at 450 nm on a spectrophotometer (XFluor4 Version: V4.51). At least three independent experiments were performed in quadruplicate. The half-maximal inhibitory concentration (IC50) values were calculated by nonlinear regression analysis using the GraphPad Prism software (Version 5.0, GraphPad Software, San Diego, CA, USA).

### Annexin V-PI flow cytometry

SKOV3-TR30 cells were plated in 6-well plates (2 × 10^5^ cells/2 mL medium). 48 h after transfection, the cells were treated with 200 nmol/L paclitaxel. After an additional 24 h, the cells were harvested and washed twice in PBS for the detection of cell apoptosis using an Annexin V-FITC Apoptosis Detection kit (BD Pharmingen, Mountain View, CA, USA) according to the manufacturer's instructions. The cells were stained with Annexin V and PI in the binding buffer for 30 min in the dark and analyzed by flow cytometry (BD FACSAria ^TM^ Fusion). Approximately 10^4^ cells were estimated per sample, and three independent experiments were performed.

### Statistical analysis

All values were expressed as mean ± standard error (SE). Statistical comparisons among different groups were performed using Student's t-test. For patient samples, the result of NF-κB1 and ELK1 mRNA expression was non-normal distribution. Therefore, we used the Mann-Whitney U test. The c-Rel mRNA expression showed a normal distribution, and hence, unpaired t-test was employed. The correlations of NF-κB1, c-Rel, and ELK1 with miR-134 in the clinical specimens were calculated using the Spearman's correlation coefficient analysis. Kaplan–Meier survival analysis and Cox proportional hazards regression model were applied to determine the survival time and prognosis factor. All statistical analyses were performed using SPSS19.0 statistical software. P-values less than 0.05 were considered to indicate statistical significance.

## SUPPLEMENTARY MATERIALS FIGURES AND TABLES




