# Supplementary material for: NF-κB1, c-Rel, and ELK1 inhibit miR-134 expression leading to TAB1 upregulation in paclitaxel-resistant human ovarian cancer
Source: Oncotarget. 2017 Feb 11;8(15):24853–68. doi: 10.18632/oncotarget.15267 (PMC5421894; doi:10.18632/oncotarget.15267)
Supplement: Supplementary file 1 [file oncotarget-08-24853-s001.pdf]

NF-κB1, c-Rel, and ELK1 inhibit miR-134 expression leading to TAB1 upregulation in paclitaxel-resistant human ovarian cancer

SUPPLEMENTARY FIGURE AND TABLES

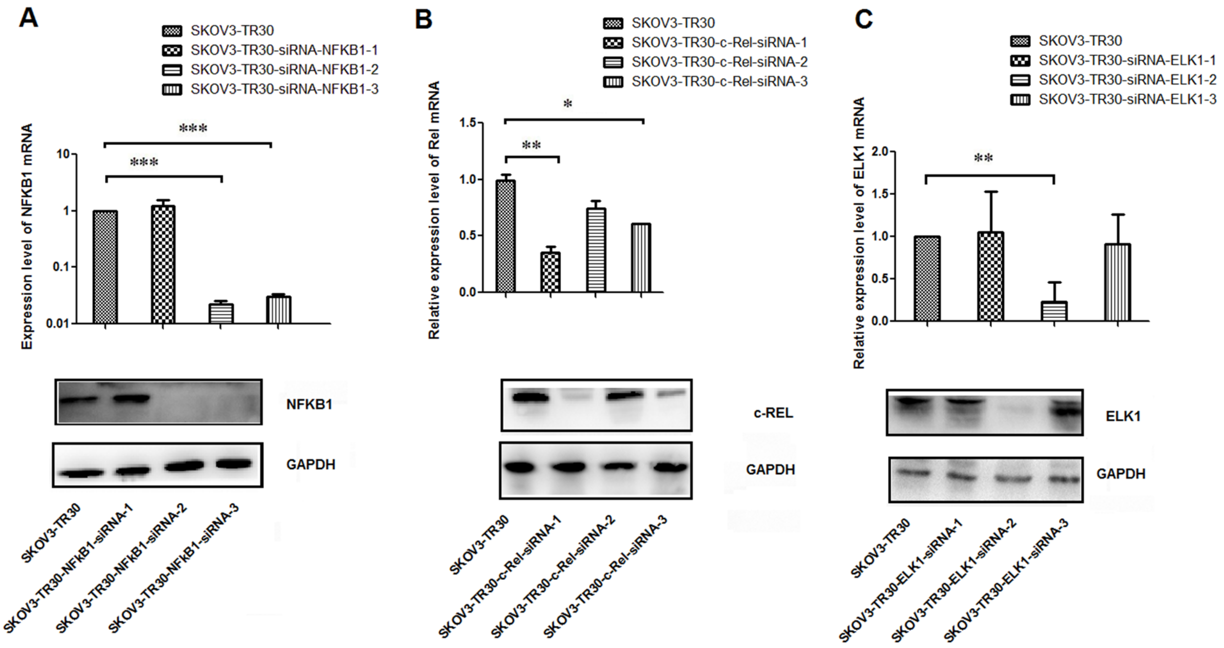

Supplementary Figure 1: Implication of NF-κB1, c-Rel, and ELK1 in regulating miR-134 expression. SKOV3-TR30 cells were transfected with siRNAs specific for NF-κB1, c-Rel, and ELK1 along with miR-134 inhibitor. 48 h post-transfection, the total RNA was extracted, reverse-transcribed, and quantified by real-time PCR. **A–C.** Transfection efficiency of siRNAs was validated by qRT-PCR and Western blot analyses. (*P < 0.05, **P < 0.01, ***P < 0.001).

Supplementary Table 1: Clinicopathologic features of 48 cases of serous EOC patients used for mRNA expression

	Chemo-resistant tissue group			Chemo-sensitive tissue group		
	WHO histological classification	Clinical Stage (FIGO 2009)	Age (y)	WHO histological classification	Clinical Stage (FIGO 2009)	Age (y)
1	Well	IIIC	42	Poorly	IIIB	54
2	Moderately	IIC	48	Poorly	IIIC	60
3	Poorly	IIIB	62	Moderately	IIIC	59
4	Poorly	IIIC	58	Poorly	IIIC	52
5	Moderately	IC	46	Poorly	IIIC	53
6	Poorly-Moderately	IIIC	51	Moderately	IIIC	57
7	Moderately	IIIB	47	Moderately	IIIA	46
8	Poorly	IIB	56	Poorly	IIIC	57
9	Moderately	IIIC	50	Moderately	IA	79
10	Poorly	IIIC	65	Moderately	IIIC	56
11	Poorly-Moderately	IIIC	63	Poorly	IIIC	58
12	Moderately	IIIC	42	Moderately	IA	33
13	Moderately	IIIB	48	Poorly	IIB	71
14	Poorly-Moderately	IIIC	48	Moderately	IIIC	62
15	Poorly	IIB	79	Poorly	IIIB	53
16	Moderately	IIIC	53	Poorly	IIIA	46
17	Poorly	IIIA	39	Poorly-Moderately	IIIC	42
18	Moderately	IIIC	64	Moderately	IIIA	41
19	Poorly	IIB	45	Moderately	IIA	57
20	Poorly-Moderately	IIC	53	Poorly	IIB	51
21	Poorly	IIB	56	Poorly	IIB	54
22	Moderately	IC	46	Moderately	IB	47
23	Poorly-Moderately	IIIC	51	Poorly-Moderately	IIIC	55
24	Moderately	IIIB	47	Moderately	IIIC	52

Supplementary Table 2: Primers for ChIP, EMSA assays, luciferease reporter construction, overexpression plasmid construction and qPCR detection

See Supplementary File 1

Supplementary Table 3: Potential targets of miR-134 identified by computational methods (TargetScan, miRDB and miRanda databases)

GeneSymbol	TargetScan	miRanda	miRDB
ARL4D	1	1	1
BACH1	0	1	1
BAGE5	1	1	1
C11ORF58	0	1	1
CALCOCO2	1	1	1
CPS1	1	1	1
CREB1	1	1	1
CUTC	0	1	1
FAM168B	1	1	1
FAM91A1	1	1	1
GRIK2	1	1	1
HNMT	1	1	1
ILDR2	1	1	1
KDM6A	1	1	0
LMLN	1	1	1
LSM12	1	1	1
MED13	1	1	1
MFAP3L	1	1	1
MRFAP1	1	1	0
MRS2	1	1	1
NIPA1	1	1	1
PHLPP2	1	1	1
PPP1R7	1	1	1
RAB27A	1	1	1
SFXN1	0	1	1
SH2B3	1	1	1
SLC30A4	1	1	1
STAT5B	1	1	1
TAB1	1	1	1
TCF21	1	1	1
USP9X	1	1	1
WDFY1	0	1	1
ZDHHC9	0	1	1
ZMAT5	1	1	1
ZSCAN12	1	1	1
