# Supplementary material for: NF-κB1, c-Rel, and ELK1 inhibit miR-134 expression leading to TAB1 upregulation in paclitaxel-resistant human ovarian cancer
Source: Oncotarget. 2017 Feb 11;8(15):24853–68. doi: 10.18632/oncotarget.15267 (PMC5421894; doi:10.18632/oncotarget.15267)
Supplement: Supplementary file 2 [file oncotarget-08-24853-s002.docx]

**Supplementary table 2**

**Primers for ChIP, EMSA assays, luciferease reporter construction, overexpression plasmid construction and qPCR detection.**

| 5'-CGACGCGT TGATTGACATTGTGCACCCCTCAAC-3' | LUC-R1 sense |
| --- | --- |
| 5'-CCGCTCGAG ATTCATCTTACCAAGGGTGAGGGTC-3' | LUC-R1 antisense |
| 5'-CGACGCGT CAGTGAATGAAATCAAGTTCTTGCT-3' | LUC-R3 sense |
| 5'-CCGCTCGAG ACTGCCCTGGGTAGATTCTCTATCA-3' | LUC-R3 antisense |
| 5'-CGACGCGT ACTTGAAGAGAAGTTGTTCGTGGTG-3' | LUC-R5 sense |
| 5'-CCGCTCGAG GACGGTGCTGACACCAACATCTCTT -3' | LUC-R5 antisense |
| 5’-ACGAATCATACAGGGAAGCTTAGTTTTTCAGTATCAAATAC-3’ | LUC-R1 -mut sense |
| 5’-GTATTTGATACTGAAAAACTAAGCTTCCCTGTATGATTCGT-3’ | LUC-R1 -mut antisense |
| 5’-CAAGGCCCATGGAAATAATACTGGCATCTTTAAGAA AAAGC-3’ | LUC-R3 -mut sense |
| 5’-GCTTTTTCTTAAAGATGCCAATATTATTTCCATGGGCCTTG-3’ | LUC-R3 -mut antisense |
| 5’-TCTTTGTGTCAACCATGTATCTCAGCCAAGAATGGATGG-3’ | LUC-R5 -mut sense |
| 5’-CCATCCATTCTTGGCTGAGATACATGGTTGACACAAAGA-3’ | LUC-R5 -mut antisense |
| 5'-TGATTGACATTGTGCACCCCTCAAC-3' | CHIP-R1-sense (500bp) |
| 5'- ATTCATCTTACCAAGGGTGAGGGTC -3' | CHIP-R1-antisense |
| 5'-CTCTGGAGACATGCTGGCTCGGTAG-3' | CHIP-R2-sense (500bp) |
| 5'-CTCTCTGCTAAGGTCCAGAATCACC-3' | CHIP-R2-antisense |
| 5'-CAGTGAATGAAATCAAGTTCTTGCT -3' | CHIP-R3-sense (500bp) |
| 5'-ACTGCCCTGGGTAGATTCTCTATCA -3' | CHIP-R3-antisense |
| 5'-GATGGGGAACCTACCAGGAGCCTGG-3' | CHIP-R4-sense (500bp) |
| 5'-ACCACAGAAAAGCAGAGACAAGACA -3' | CHIP-R4-antisense |
| 5'-ACTTGAAGAGAAGTTGTTCGTGGTG-3' | CHIP-R5-sense (359bp) |
| 5'-GACGGTGCTGACACCAACATCTCTT -3' | CHIP-R5-antisense |
| 5' BIOTIN-CATACAGGGACATCCAGTTTTTCA-3' | ELK1 EMSA oligo sense strand |
| 5' BIOTIN-TGAAAAACTGGATGTCCCTGTATG-3' | ELK1 EMSA oligo antisense strand |
| 5' BIOTIN-GGCCCATTTCCCGCCCCCTGGCAT-3' | NF-κB1 EMSA oligo sense strand |
| 5' BIOTIN-ATGCCAGGGGGCGGGAAATGGGCC-3' | NF-κB1 EMSA oligo antisense strand |
| 5' BIOTIN- ACCATGTGGAAAAGCCAAGAATGGA -3' | c-Rel EMSA oligo sense strand |
| 5' BIOTIN- TCCATTCTTGGCTTTTCCACATGGT -3' | c-Rel EMSA oligo antisense strand |
| 5'- CATACAGGGACATCCAGTTTTTCA-3' | ELK1 EMSA oligo sense strand (unlabled) |
| 5'-TGAAAAACTGGATGTCCCTGTATG-3' | ELK1 EMSA oligo antisense strand (unlabled) |
| 5' -GGCCCATTTCCCGCCCCCTGGCAT-3' | NF-κB1 EMSA oligo sense strand (unlabled) |
| 5' -ATGCCAGGGGGCGGGAAATGGGCC-3' | NF-κB1 EMSA oligo antisense strand (unlabled) |
| 5' -ACCATGTGGAAAAGCCAAGAATGGA-3' | c-Rel EMSA oligo sense strand (unlabled) |
| 5' -TCCATTCTTGGCTTTTCCACATGGT-3' | c-Rel1 EMSA oligo antisense strand (unlabled) |
| 5’-CGACGCGT ATGGCAGAAGATGATCCATATTTGG-3’ | NF-κB1 inner sense |
| 5’-CCCAAGCTT CTAAATTTTGCCTTCTAGAGGTCCT-3’ | NF-κB1 inner antisense |
| 5’-GCCGCCGCCTCTTCCTTCTCCAGCC-3’ | NF-κB1 outer sense |
| 5’-CGCAGTGGAATTTTAGGG-3’ | NF-κB1 outer antisense |
| 5’-CGACGCGT ATGGACCCATCTGTGACGCTGTGGC-3’ | ELK1inner sense |
| 5’-CCCAAGCTT TCATGGCTTCTGGGGCCCTGGGGAG -3’ | ELK1 inner antisense |
| 5’-AGCCTCAACTTTCAGGAGACCCGTC-3’ | ElK1 outer sense |
| 5’- TGGGGGACTGACAGAAAACAGAGAA-3' | ELK1 outer antisense |
| 5’-CGACGCGT ATGGCCTCCGGTGCGTATAACCCGT -3’ | c-Rel inner sense |
| 5’-CCCAAGCTT TCCCCAGTTAGACAAATACA-3’ | c-Rel inner antisense |
| 5’-CCTCGGCCTCCTGACTGACTGACTG-3’ | c-Rel outer sense |
| 5’-TCCCCAGTTAGACAAATACA-3’ | c-Rel outer antisense |
| 5’-GAGGCGGGCGCTCCCGCAGGGGTTC-3’ | TAB1 outer sense |
| 5’-CAGACCAGGCGACAAAAC-3’ | TAB1 outer antisense |
| 5’-CGACGCGT TGGCGGCGCAGAGGAGGAGCTTGC-3’ | TAB1 inner sense |
| 5’-ACGCGTCGAC TACCCTGGGGTCAGGCTGCCCAGGA-3’ | TAB1 inner antisense |
| 5’-GGTGAGAGGATTTAAAGTACTCAGTAAAGGCTTGGGAACAA -3’ | TAB1-mut sense |
| 5’-TTGTTCCCAAGCCTTTACTGAGTACTTTAAATCCTCTCACC-3’ | TAB1-mut antisense |
| 5’-GGACTAGT CAGGGCCTGGTGTGGAACCAGGGA-3’ | TAB1 sense |
| 5’-CCCAAGCTT AAACAGCACCACAGAGGC-3’ | TAB1 antisense |
| 5’-TCAGCAGGCAGCATTCCA-3’ | c-Rel sense for qPCR |
| 5’-CGTTCTTGTCCAAATTCTGCTTCA-3’ | c-Rel antisense for qPCR |
| 5’-GTGAGGATGGGATCTGCACTGTA-3’ | NF sense for qPCR |
| 5’-GGTGCACCAAGAGTCCAGGATTA-3’ | NF antisense for qPCR |
| 5’-TTCCTGTACATCGTAGCGAATCAA-3’ | ELK1 sense for qPCR |
| 5’-TCAGCAGCTGCAGCAGAAAC-3’ | ELK1 antisense for qPCR |
| 5’-GTGACGGGCTTCTTGGTGCT-3’ | TAB1 sense for qPCR |
| 5’-CTTGGCAAACTCAGTGTCAATCATC-3’ | TAB1 antisense for qPCR |
| 5'-GTACGACTCACTATAGGGA AGGTCCACCACCCTGTTGCTGT-3' | GAPDH sense |
| 5'-AGGTGACACTATAGAATAAACAGCGACACCCACTCCTCCA-3' | GAPDH antisense |
| 5'-CAAUUUCCCACACCGUGUATT-3' | siRNA-NF-κB1-1 sense |
| 5'-UACACGGUGUGGGAAAUUGTT-3' | siRNA-NF-κB1-1 antisense |
| 5'-CUUAUGGUGGGAUUACUUUTT-3' | siRNA-NF-κB1-2 sense |
| 5'-AAAGUAAUCCCACCAUAAGTT-3' | siRNA-NF-κB1-2 antisense |
| 5'-CAGAGUUUACAUCUGAUGATT-3' | siRNA-NF-κB1-3 sense |
| 5'-UCAUCAGAUGUAAACUCUGTT-3' | siRNA-NF-κB1-3 antisense |
| 5'-CUGAUAAUCCAAGACUUCUTT-3' | siRNA-c-Rel-1-sense |
| 5'-AGAAGUCUUGGAUUAUCAGTT-3' | siRNA-c-Rel-1-antisense |
| 5'-CAUAUAAACCUCAUCCUCATT-3' | siRNA-c-Rel-2-sense |
| 5'-UGAGGAUGAGGUUUAUAUGTT-3' | siRNA-c-Rel-2-antisense |
| 5'-CAUCAGAUGUCCUCUUCCATT-3' | siRNA-c-Rel-3-sense |
| 5'-UGGAAGAGGACAUCUGAUGTT-3' | siRNA-c-Rel-3-antisense |
| 5'-GCUUCCACAACUUCUCUGUTT-3' | siRNA-ELK1-1-sense |
| 5'-ACAGAGAAGUUGUGGAAGCTT-3' | siRNA-ELK1-1-antisense |
| 5'-GGGUUUGUGCCAGAAACCATT-3' | siRNA-ELK1-2-sense |
| 5'-UGGUUUCUGGCACAAACCCTT-3' | siRNA-ELK1-2-antisense |
| 5'-CUCCCAAGAGUAACUCUCATT-3' | siRNA-ELK1-3-sense |
| 5'-UGAGAGUCACUCUUGGGAGTT-3' | siRNA-ELK1-3-antisense |
| 5'-CUGCCAAGUCCAAACCAAUTT-3' | si-RNA-TAB1-sense |
| 5'-AUUGGUUUGGACUUGGCAGTT-3' | si-RNA-TAB1-antisense |
|  |  |
